# Tiger Nut (*Cyperus esculentus*) as a Functional Ingredient in Gluten-Free Extruded Snacks

**DOI:** 10.3390/foods9121770

**Published:** 2020-11-29

**Authors:** Nicola Gasparre, James Pan, Priscila Leal da Silva Alves, Cristina M. Rosell, Jose De J. Berrios

**Affiliations:** 1 Western Regional Research Center, U.S. Department of Agriculture Research Service, 800 Buchanan Street, Albany, CA 94710-1105, USA; nicgasp@iata.csic.es (N.G.); james.pan@usda.gov (J.P.); priscila.alves@usda.gov (P.L.d.S.A.); 2 Food Science Department, Institute of Agrochemistry and Food Technology (IATA-CSIC), C/Agustin Escardino 7, 46980 Paterna, Spain; crosell@iata.csic.es

**Keywords:** tiger nut, rice flour, gluten-free, snacks, extrusion

## Abstract

Tiger nut (TN) is a nutritious source of gluten-free flour, used generally in healthy beverages, but its incorporation in gluten-free extruded snacks has not been explored. TN flour was blended at different concentrations (up to 70%) with rice flour and soluble fiber, for the development of gluten-free snacks on a twin-screw extruder. The effect of TN inclusion in the formulations was evaluated on relevant physiochemical characteristics of the snacks. Viscoamylograph of the raw formulations showed that TN addition increased (*p* < 0.01) onset temperature and delayed peak viscosity. In the extruded flours, TN contributed to limit the starch degradation during extrusion. Diameter, expansion ratio, true density, and total pore volume of the extrudates were reduced (*pf* < 0.01) by the increased TN content in the formulations, while bulk density rose. The surfaces of the extruded snacks were modified by the increasing inclusion of TN in substitution of rice in the formulations. Extrudates containing 10% TN showed the best overall texture profile. Moreover, TN addition enhanced the ash and protein content of the snacks and increased their total antioxidant activity. This study demonstrated that incorporation of 10% TN flour into rice-based formulation was suitable for making gluten-free snacks with acceptable physical properties.

## 1. Introduction

In the last twenty years, patients diagnosed with celiac disease have represented an important public health problem [1]. This event has prompted an increase in demand for gluten-free (GF) food products by consumers with and without celiac disease. In fact, many non-celiac individuals wrongly believe that a GF diet is an essentially healthier choice [2]. The market demand of GF foods has promoted the need for new research to develop products using GF agricultural commodities and novel technologies. Extrusion cooking is a versatile technology that has found dominant uses in the cereal and pet food industries as well as in dairy, bakery, and confections industries. In general, the final extrudate has low moisture content and considered a shelf-stable food product [3]. Extrusion process promotes starch gelatinization, protein denaturation, lipid oxidation and the formation of new complexes occur as result of macromolecules interaction which contribute to changes in microstructure [4] and color [5]. In the last decade, studies have been carried out to develop expanded GF snacks, made mainly from cereal-based mixes, to provide nutritious GF foods to consumers inflicted with celiac disease [6,7]. Most recently, other flour mixes containing unconventional crops and agriculture by-products, such as passion fruit shell, and rice flours [8], plantain and chickpea flours in a corn-based mix [6], blends of apple pomace, corn and sorghum [7] and amaranth, quinoa and kañiwa [9] were also evaluated for the development of GF foods. The interest in alternative agricultural commodities is still increasing.

Tiger nut (*Cyperus esculentus*) (TN) is an underutilized crop with great potential for the development of a variety of value-added, nutritious GF foods, including ready-to eat extruded snack-type products. TN is a tuber generally cultivated in the Eastern region of Spain and Western part of Africa [10]. Their rhizomes are used in some countries for human consumption in various forms. In Spain, TN is only grown and processed into a popular drink called “horchata de chufa” in the province of Valencia. “Horchata” is also gaining popularity in other countries due to numerous health related benefits. The composition of TN is characterized by high contents of insoluble fiber and unsaturated fat and with relatively low concentration of starch [11]. Literature on the use of TN in GF food formulation is limited. Aguilar et al. [12], used a combination of TN and chickpea flour to replace emulsifier and/or shortening partially or totally in GF batters or doughs and breads formulations, and reported acceptable specific volume and darker crust in the GF products. Demirkesen et al. [13] reported that TN flour added up to 25% to the rice flour increased the gelatinization temperatures in GF bread, while a significant reduction of the onset gelatinization temperature and peak temperature was observed when TN flour was added to corn-based biscuits [14]. Gasparre et al. [10], reported the effect of a selection of hydrocolloids on the dough rheology, texture properties and cooking performances of GF noodles made by TN. Only one study has reported using TN as food ingredient to produce extrudates [15]. The authors found the mixes of TN-cassava used in their study difficult to extrude as the TN concentration increased in the mixes, due to the high content of insoluble fiber and fat present in TN [16], that caused pressure drop in the extruder barrel and reduced expansion. Moreover, focusing on the sensory quality of GF cereal-based foodstuffs, TN incorporation (20%) produced the highest overall acceptability scores of corn-made biscuits [17]. It is known that insoluble fiber reduce expansion, while soluble fiber tends to increase the expansion of the extrudate [18], and that fat content can cause slippage of melt into the barrel with consequent low pressure at the die exit [19]. The aim of the present study was to develop GF extruded snacks from TN flour/rice flour mixes and the evaluation of nutritional, physical, and microstructural qualities of the extrudates. 

## 2. Materials and Methods

### 2.1. Raw Materials

Tiger nut (TN) flour, short grain rice flour (Koshihikari, amylose: 17.6%) and Nutriose^®^ were used for making the extruded snacks. TN flour had the following proximate composition: 26.3% fat 18.3% fiber and 8.5% of protein, as declared on the nutritional label, was provided by Mon Orxata (Valencia, Spain). Short grain rice was provided by a local rice grower (Richvale, CA, USA) and Nutriose^®^ FM06, a plant derived soluble fiber (SF) with neutral taste, was supplied by Roquette Company (Geneva, IL, USA).

### 2.2. Tiger Nut Milling and Blends Preparation

Preliminary studies were carried out to mill the TN into flour by overcoming the problem related to its high fat and fiber content. Two different mills were tested to archive this goal. A laboratory Cyclone mill (Udy Corp., Fort Collins, CO, USA) fitted with a 0.5-mm screen and chilled grinding surface. Then, a comminuting mill fitted with a 3 mm screen (Model D, the Fitzpatrick Company, Chicago, IL, USA), which gave the best result. To further reduce the particle size of the obtained coarse flour and avoid stickiness of the TN to the milling surface, due to its high fat content, the coarse flour mixed with dry ice (3:1) and milled on a Wiley mill (Arthur H. Thomas Co, Philadelphia, PA, USA) fitted with a 2 mm mesh. The resulting flour was a finer flour with a mean particle size below 200 µm. Short grain rice was milled in a pin mill (model 160Z, Micron Hosakawa, Köln, Germany) to a fine flour. TN flour was mixed in increasing proportions, 10%, 30%, 50% and 70% with rice flour and 10% SF, which represented formulations, referred from here on as, R1, R2, R3 and R4, respectively. A mix of 90% rice and 10% SF was used as a control, which allowed increasing the fiber content of the final products. The blends were stored at 4 °C until extrusion cooking was carried out.

### 2.3. Modified Feeding Device and Extrusion Processing Conditions

#### 2.3.1. Modified Feeding Device

**Figure 1:**
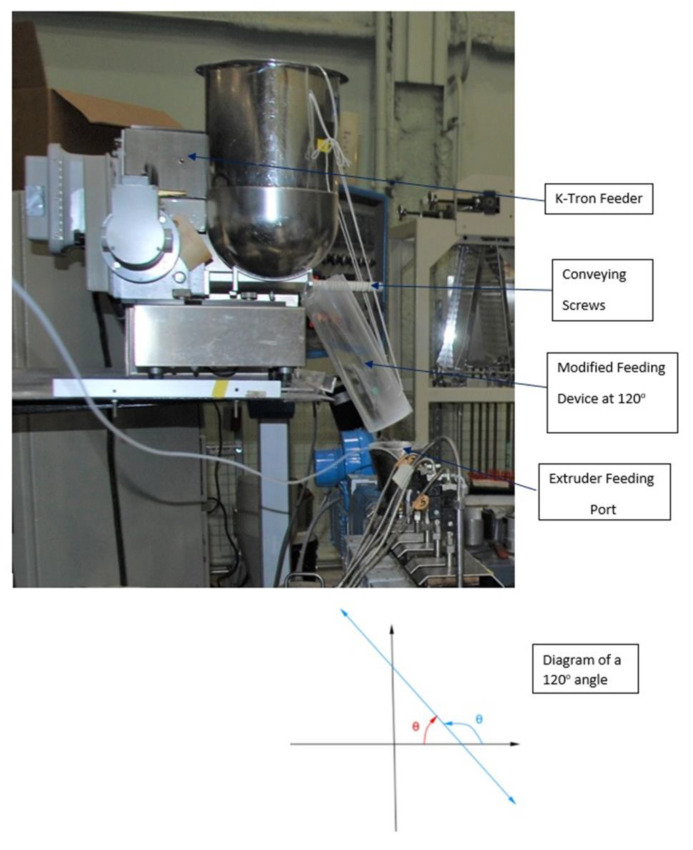
Modified feeding device for suitable conveying formulated flours, containing different levels of tiger nut, into the extruder.

The high fat content in the TN flour made the formulated flours difficult to freely flow through the twin screw of the K-Tron feeder (Model KCL-24-KT-20, K, K-Tron Corp., Pitman, NJ, USA) used in this study, to deliver the correct amount of feed into the extruder. To enable their proper flow, a feeding modification system was built, which consisted of a 4 inches acrylic tube (Tap Plastic, San Leandro, CA, USA) cut lengthwise. The surface of the channel was coated twice with an anti-static solution (food-grade) containing fatty acid in its composition. The made flour conveyor was suspended underneath the feeder discharge chute and adjusted to provide an inclination of about 120 degrees. This modification allowed the proper flow of flour, to the set feed targets of flour, to be delivered into the extruder’s feeding port (Figure 1).

#### 2.3.2. Extrusion Processing Conditions

**Figure 2:**
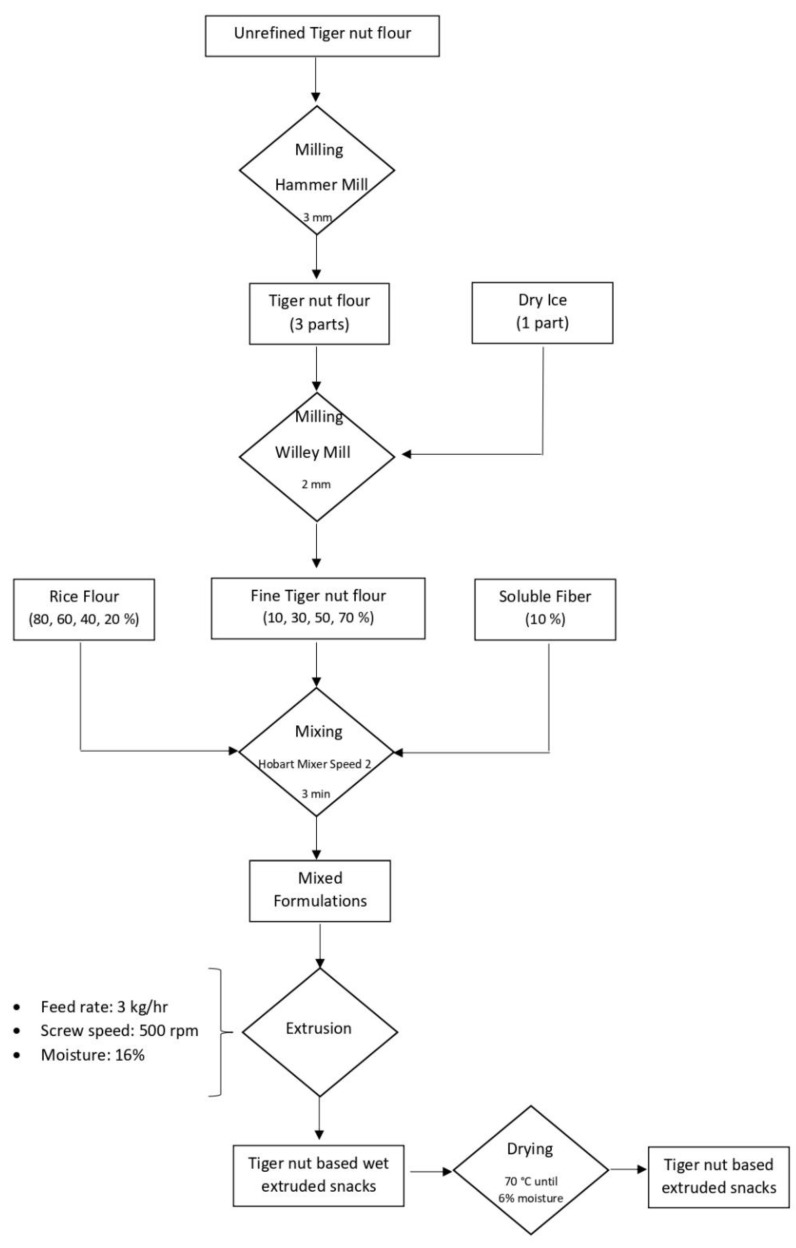
Flowchart of snacks production from tiger nut/rice flour mixture.

Extrusion of the formulated flours was performed using the Leistritz 18 mm co-rotating twin-screw extruder (MIC 18/GL 30D, Allendale, NJ, USA), equipped with six heating-cooling barrel zones. The heating profile of the barrel zones were set at the following temperatures: zone 1 cooled with tap water at approximately 25 °C, zone 2 at 60 °C, zone 3 at 80 °C, zone 4 at 100 °C, zone 5 at 100 °C and zone 6 at 120 °C. The first temperature was the temperature of the feeding zone and the last one corresponded to the temperature of the die zone (expansion zone). The die was a circular orifice 3 mm in diameter. During the extrusion process, the feed rate and the screw speed were kept constant at 3 kg/hour and 500 rpm, respectively, based on preliminary testing. During extrusion, the moisture content of the formulations was adjusted to 16% by injecting water through a preparatory HPLC pump (Model 305, Gilson, Middleton, WI, USA). The extrudates obtained were dried at 70 °C in a force air drying oven (Imperial IV, Labline, Melrose Park, IL, USA) to a final moisture content of 6%. Extrudates from formulations containing 10%, 30%, 50% and 70% TN flour were referred as E1, E2, E3 and E4, respectively. The dried extrudates were ground by a laboratory sample mill (Cyclone Mill, Udy Corporation, Fort Collins, CO, USA) fitted with a 0.5 mm mesh screen into fine flour for further evaluation. Figure 2 illustrates the detailed steps in obtaining TN extrudates from the raw ingredients.

### 2.4. Proximate Analysis

Proximate composition of raw and extruded flours was determined in triplicate according to standard methods of the Association of Official Analytical Chemist AOAC [20]. Total nitrogen was determined by Leco FP628 (Leco Company, St. Joseph, MI, USA), and fat content determined on a Dionex ASE350 solvent extractor (Thermo Fisher Scientific, Inc., Waltham, MA, USA). Carbohydrate content was calculated by difference.

### 2.5. Apparent Viscosity

Apparent viscosity of the raw blends and extruded flours was determined using a Rapid Viscosity Analyzer (RVA 4500, Perten Instrument, Sydney, NSW, Australia). Samples of 3.50 g and 25.0 mL HPLC-grade water were mixed in an RVA canister and analyzed using the following temperature profiles: equilibrating at 25 °C for 1 min, then the temperature was ramp up to 95 °C over a period of 3 min, and holding at 95 °C for 4 min, followed by a cooling period of 3 min to 25 °C, and hold for 2 min. Thermocline software for Windows (TCW3) was used to calculate the pasting parameters: onset temperature where viscosity start to increase; peak viscosity (Pa·s): the maximum hot paste viscosity; trough viscosity (Pa·s): minimum hot paste viscosity; breakdown (Pa·s): the difference between peak viscosity and trough viscosity; final viscosity (Pa·s): the viscosity at the end of the run; and total setback (Pa·s): the difference between final viscosity and trough viscosity.

### 2.6. Microstructure Analysis

A scanning electron microscope (SEM) (Model TM300, Hitachi High-Technologies, Tokyo, Japan) at 2 kV accelerating voltage and at magnification of 100× was used for microstructural analysis. Extrudates were dried overnight in a desiccator with CaSO_4_ prior to analysis. Colloidal graphite cement was applied around the bottom of the extrudates for a better conductivity and covered with approximately 30 nm of gold using a Technics Hummer V sputter coater. Stereoscopic pictures were taken using a stereoscopic microscope (Leica Microsystems Inc., Buffalo Grove, IL, USA.) at 1× magnification.

### 2.7. Final Products Quality Parameters

Expansion ratio (ER) of the extrudates was evaluated according to the method described by Berrios et al. [21] and calculated as followed:

ER (mm) = Area of extruded road (mm^2^)/Area of circular die hole (mm^2^) (1)

The bulk density (BD) of each of the extrudates was determined based on a volumetric displacement method, using glass beads with a diameter of 2 mm as a displacement medium, with some modifications as previously explained by Patil et al. [22], who standardized this measurement with optimum sample size. The values employed were obtained by averaging five measurements of the extrudates.

BD (g/cm^3^) = Extrudate mass (g)/Extrudate volume (cm^3^) (2)

True density of extrudates was measured with a pycnometer (AccuPyc II 1340, Micromeritics, Norcross, GA, USA) using a small sample holder with a volume of 350 cm^3^. Helium was used as a volume displacement medium. To calculate sample volume and total pore volume, the pressure before and after expansion was measured. The analysis was carried out in triplicate for each sample.

A texture analysis of the extrudates was carried out using a texture analyzer (TA-XTPlus, Stable Micro System, Godalming, Surrey, UK) calibrated with a 2 kg mass, to determine the firmness and shear force of the samples by compression. A 3-point band test was used on individual extruded rods. A TA92A probe was set to press the extruded rod placed on a metal platform to a depth of 15%, with testing speed of 1 mm/s. The return distance and return speed were adjusted to 35 mm/s and 10 mm/s, respectively. A total of 20 measurements were performed for each sample. Firmness was defined as the peak force of the first compression required for the sample to rupture and the shear force was calculated as the area under the curve from the force/time graph.

### 2.8. Total Soluble Phenolics and Total Antioxidant Capacity of Final Products

Total soluble phenolics (TSP) were determined according to the Folin–Ciocalteu spectrophotometric method, with a slight modification to the method as described by Nayak et al. [23]. About 5 g of extruded flour samples were extracted with 20 mL of methanol at room temperature (25 °C) for 24 h. About 0.15 mL of Folin–Ciocalteu reagent was added to the extract (0.3 mL of aliquot). The mixture was set aside to equilibrate for 3 min and then mixed with 0.3 mL sodium carbonate. Subsequently, incubated at room temperature for 60 min, and absorbance of the mixture was read at 765 nm with a benchtop spectrophotometer (PharmaSpec UV-1700, Shimadzu Scientific Instruments, Inc., Kyoto, Japan). Methanol was used as a blank. TSP content was quantified from a gallic acid standard curve developed from 0–0.125 mg of gallic acid per mL and expressed as micrograms of gallic acid equivalent per milligrams of dry weight sample (μg GAE/mg DW). The analysis was carried out in triplicates.

Total antioxidant capacity (TAC) of extruded samples was measured using an adapted method of Patel et al. [24]. The methanol extracts from the TSP were used in this evaluation. A total of 0.5 mL of sample solution was reacted with 2.95 mL of 2,2-diphenyl-1-picrylhydrazyl (DPPH) (103.2 μM in methanol, absorbance of ~1.2 at 515 nm) on a covered shaker at room temperature for 20 h, and spectrophotometer was blanked with methanol. The absorbance was read at 515 nm. TAC was quantified from a Trolox standard curve developed from 0 to 750 µg of Trolox per mL and expressed as micrograms of Trolox equivalent per gram of dry weight sample (μg TE/g DW). The analysis was carried out in triplicates.

### 2.9. Statistical Analysis

Data were analyzed by multifactor analysis of variance (MANOVA) using Statgraphics Centurion XVII software (Statpoint Technologies, Warrenton, VA, USA). Fisher’s least significant differences test was applied for the determination of significant differences among experimental mean values, with 95% confidence.

## 3. Results and Discussion

### 3.1. Raw Blends Characteristics

The moisture and carbohydrate content of raw blends decreased significantly (*p* < 0.01) whereas the protein, fat and ash contents increased (*p* < 0.01) as the TN content was increased (Table 1). Protein, fat and ash content significantly (*p* < 0.01) increased with the TN level substitution. In fact, this trend is attributed to a higher protein, fat and ash content of TN. The same trend was observed in the study by Kareem et al. [15], where the addition of TN (up to 100%) to a mixture of cassava and spices greatly enhanced the ash, protein, and fat content of the raw blends. 

**Figure 3:**
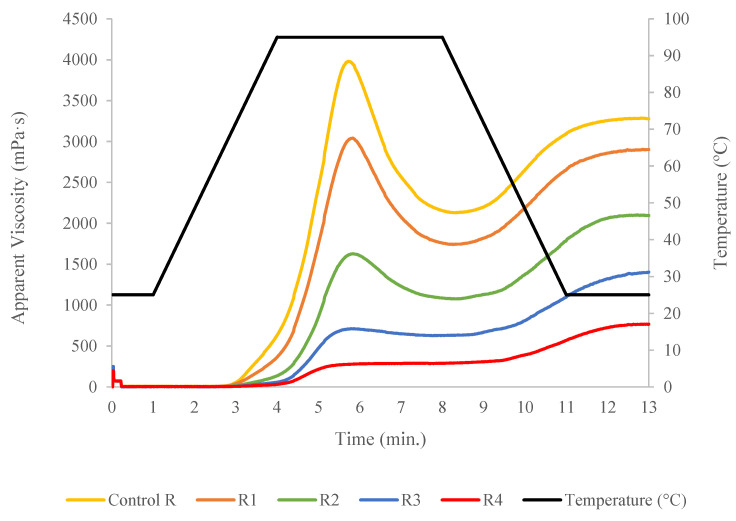
Rapid Visco Analyzer profiles of formulated unprocessed flours containing different concentration of tiger nut flour.

The Rapid Visco Analyzer (RVA) was used to visualize the physical transition points by recording the apparent viscosity of the formulated flours. Figure 3 depicts the viscoamylographs of the raw blends. Attributes determine from the recorded apparent viscosity are exhibited in Table 1, and the presence of TN was identified as main factor of variance. Statistical analysis indicated that TN incorporation in the formulations significantly (*p* < 0.01) increased the onset temperature for samples R3 and R4 (Table 1). The blend with the highest TN replacement level (R4) required more time to achieve the maximum peak viscosity (*p* < 0.01). This delay may be caused by the presence of more quantity of fiber and fat that could limit the water required for starch swelling. TN flour produced a significant (*p* < 0.01) reduction in the peak viscosity, trough viscosity, breakdown, final viscosity, and total setback. These outcomes were related with the lower starch content in the blends with TN flour that caused a viscosity drop in the mixture, which might constraint the further expansion of the extrudates. A similar trend was observed in a previous study by Adegunwa et al. [25] when TN was added to plantain flour.

### 3.2. Extruded Products Characteristics

**Figure 4:**
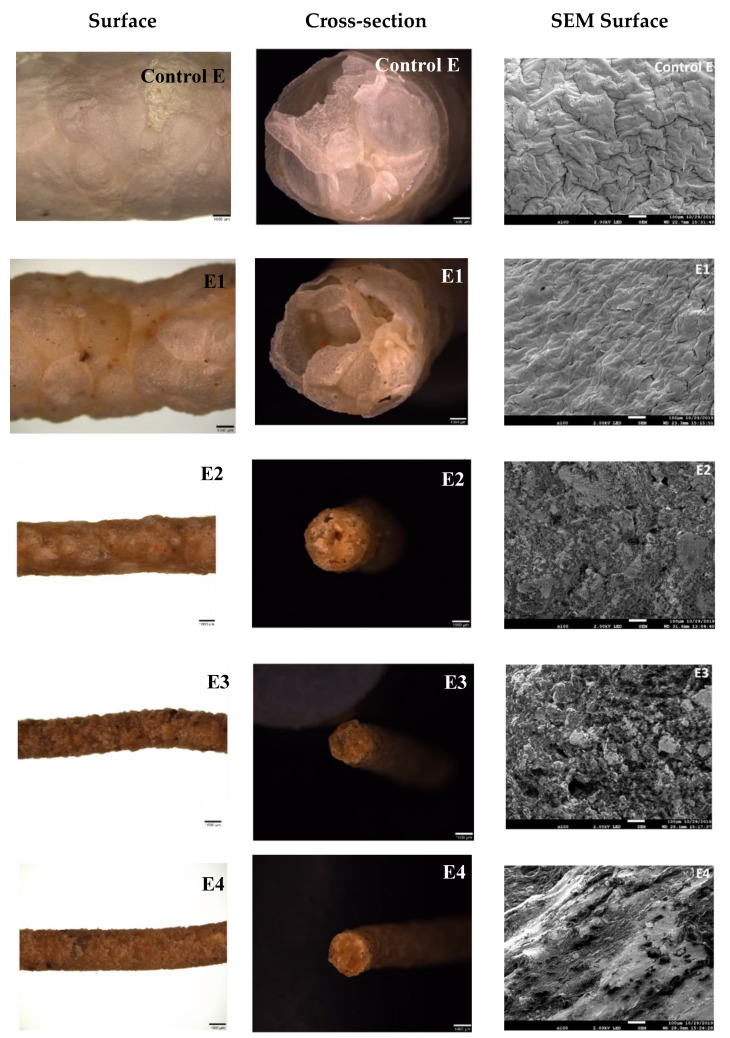
Stereoscopic images of surface and cross-sectional areas (1× magnification) and SEM micrographs (100× magnification) of the surface area of extruded samples containing different levels of tiger nut. Bottom bars in the pictures are referred to magnification, which is described above for better understanding.

Expansion of the extrudates was significantly affected by the level of TN, as it can be observed in the surface and cross section images (Figure 4). The control rice extrudate showed a corrugated, cohesive overlapping surface that was more visible in the SEM micrograph, which is attributed to the gelatinization starch and expansion of the product during the extrusion process. The cross-section of the control sample (Figure 4, Control E), which had the greater expansion, depicted larger air pockets than samples containing TN. The extrudate containing 10% TN in the formulation (Figure 4, E1), presented a smoother surface with less obvious grooves, with a reduced diameter and expansion ratio; this is due to a content reduction of starchy rice as a result of high fat TN flour incorporation. With increasing TN concentration up to 30% in the formulations, the cohesiveness and continuity of the structural surface of the extrudates are disrupted and more uneven surfaces with porous structures were observed, particularly in SEM micrograph (Figure 4, E2). This disruption is more noticeable with the sample containing 50% TN (Figure 4, E3), which presents a coarser surface and particle disaggregation. A reduction in starch concentration as the TN was increased in the formulations mostly induced a breakage of the continuous matrix in the extrudates, rendering products with less expansion and surface uniformity. Rupture and non-uniform surface have also been reported as negative quality attributes determined on extruded cornstarch when adding wheat fiber [26]. The surface appearance of extrudates containing the highest content of TN of 70% (Figure 4, E4) displayed irregular areas with an alternating smooth, oily-looking appearance. The high fat content in this sample could have acted as a plasticizer, which was observed in the SEM image, resulting in a product with the indicated distinctive surface characteristics. This result corroborates with the compact, unexpanded cross-sectional view of this extrudate (Figure 4, E4), confirming the significant effect of the inclusion of the TN flour in the structural and surface characteristics of the developed snacks. Apart from that, the compactness above described might be also linked to the higher protein and fiber content. During extrusion, protein and fiber could behave as a dispersed phase within the continuous starch arrangement that causes an interruption of the cell wall formation [27]. Furthermore, the covalent and nonbonding interactions between protein leads to the formation of a network. This protein system may influence the water distribution in the matrix causing changes of the extensional properties of the melt with a consequent density increase of the final extrudates [28].

**Figure 5:**
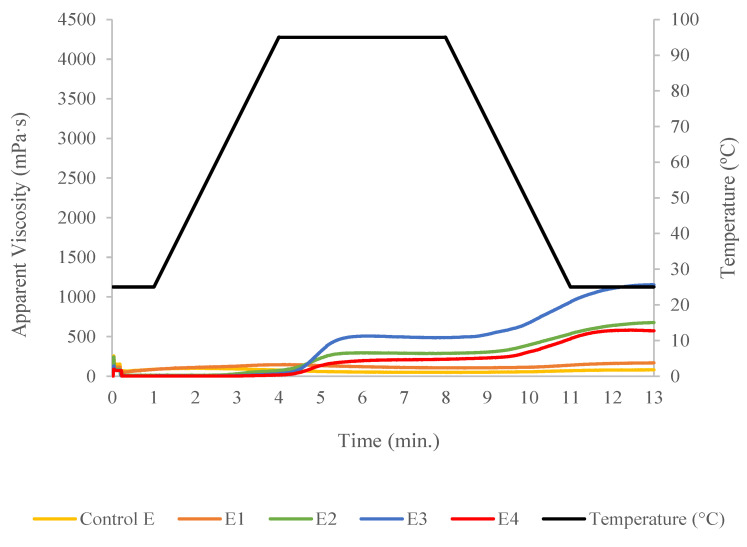
Rapid Visco Analyzer profiles of formulated extruded samples containing different levels of tiger nut flour.

Figure 5 depicts profiles of extrudates characterized by lower viscosities than those obtained for the raw blends. Indeed, heating and mechanical shearing applied during the extrusion process caused starch gelatinization. It is known that heating and mechanical shearing cause the fragmentation of amylopectin and amylose structure, thereby reducing their absorbing and swelling capacity in starchy materials [29], which was reflected in a viscosity reduction found in this study. Moreover, it was determined that the apparent viscosity was extremely low in the control and E1 extrudate containing 10% TN, than extrudates E2 and E3 containing 30% and 50% TN, respectively (Figure 5). This could be due to the “protective” effect of fat on the starch granules [30], that kept some ungelatinized granules which contribute to increase the apparent viscosity. Another explanation may be related with the starch-protein interactions that limit the starch swelling and the changes associated to its gelatinization [31]. In this case, protein absorb more water, making it less available for the complete starch gelatinization. Viscosity of extrudate E4 with 70% TN did not follow the indicated pattern, because of the greater substitution, most of or all of the starch got gelatinized and dextrinized during the extrusion process. As shown in Table 2, TN flour addition significantly (*p* < 0.01) increased the onset temperature. In this regard, formulation E4 with the highest amount of TN presented the highest onset temperature. The delay in the peak viscosity caused by TN incorporation, observed in the raw blends, was also observed when analyzing the extruded samples, while peak viscosity, trough viscosity, final viscosity and total setback showed a completely different pattern. In general, except for extrudate sample E4, the content of TN in the extruded products corresponded with a significant (*p* < 0.01) increase in the apparent viscosity, despite the reduced starch content. This may be due to the protective effect exercised by the fat molecules that could arrange themselves around the starch granules mitigating the degradation effects of the heating and mechanical shearing.

The data in Table 2 indicate that the addition of TN in the formulations produced significant (*p* < 0.01) changes in the developed extrudates. The diameters, expansion ratios, true densities, and total pore volumes progressively decreased (*p* < 0.01) as a consequence of the increased content of TN in the samples. Formulations E3 and E4 recorded the lowest values in terms of diameter and expansion ratio, as shown in Figure 4 and Table 2. The reduction in expansion with the progressively higher inclusion of TN in the samples might be attributed to a reduction in the starch content and an increase in the insoluble fiber [18], fat and protein, which are known as factors that negatively correlate with expansion [32]. In literature, studies agree with the results presented in this work, the expansion ratio decreased when TN flour was blended with rice flour [15] and also when lentil [33], mango peel [34], and partially defatted hazelnut flour, high in fiber and protein [35] were used in combination with corn flour and rice grits, respectively. Berrios et al. [21] have previously indicated that bulk density was inversely related to expansion ratio, which agrees with the results from this study. Overall, with the increase of the TN flour in the formulation a rise of the bulk density of the extrudates was observed. In particular, between the control and sample E1 there was a slight increase that was even more pronounced between the samples E2 and E4, most likely due to the excessive fat content in those samples containing more TN in their formulation. A similar trend in BD was observed by previous researchers in extrudates made with corn and soybean hull [36]. The determination of true density and porosity of the extrudates provided additional quality parameters on the properties of the developed products, as these parameters provide insight into the structural properties of the dried materials. Addition of TN significantly reduced (*p* < 0.01) the true density of the extrudates. A similar trend was observed for the total pore volume of extrudates with higher inclusion of TN in their formulation (Table 2). During extrusion, as the melt exit the die, numerous small air cells are generated by the rapid release of the high pressure [26]. The pressure difference out of the die causes the water flash off with the formation of internal pores of varying sizes that are responsible for the product expansion. The high fat content in samples with the greatest inclusion of TN in their formulation, decreased the melt viscosity of the extruded material, which caused a reduction in die pressure, resulting in less expanded products with higher bulk densities (Table 2) [37]. This phenomenon is clearly shown in Figure 4. Extrudate control E had larger diameter and more air pockets, while sample E4, with the highest amount of TN, showed a cross-section devoid of air pockets or porous structure. Internal microstructure of the extrudates are of important consideration in the production of snack-type products as this structural pore formation is an important quality parameter associated with the crispiness and crunchiness of expanded products [37].

Texture was evaluated by measuring the firmness (force required to break the extruded rod) and work of shear (area under the curve). The results in Table 2 show significant effect (*p* < 0.01) of TN on the texture of the developed extrudates. The sample with 10% of TN presented the highest firmness and shear force values. This textural effect may be due to a higher integration of the matrix components and the amylose-lipid complex formation. In fact, the interaction of fatty acids or long-chain alcohols with amylose double helices forms the amylose-lipid complexes that cause functional modifications in the physical and chemical behavior of the starch [38]. Given that, the interactions between amylose and fatty acids increase the elasticity of the starch matrix, the structure resulted to be more resistant to breakdown [39]. Conversely, both texture parameters were reduced with an increase of TN in the formulations. This could be explained by the reduction of starch content as the addition of TN increased in the formulations, while fat content greatly increased resulting in extrudates with softer and more brittle texture. This same trend was also reported in a previous study on extrudates containing different levels of cassava-TN mixtures [15]. Moreover, the outcomes about the maximum force value from this study (from 242 g to 922 g) are in the same range of those reported by Kareem et al. [15] (from 251 g to 1272 g).

The proximate analysis of the raw blends determined that the TN addition significantly (*p* < 0.01) increased the protein, fat and ash content compared to the control sample with no TN addition. It was also observed that extrusion process reduced the protein and fat amount in the extruded snacks. Protein losses ranged from 11% to 16% but those were much greater for the fat content. The control suffered the greatest fat loss followed by sample E1 (150%). This reduction of fat most likely occurred at the die opening as free oils [40]. As melt exits the die, a rapid temperature and pressure drop occur, resulting in rapid expansion of the water molecules into steam, and in this research, a small quantity of liquified fat was observed at the die, especially for the samples E4 and E5. Another possible explanation of the fat reduction could be the formation of new complexes with amylose or protein that trap the lipid, making their extraction more difficult with conventional method [40]. After extrusion, all the samples, (except the control) presented a slightly higher ash content with respect to their raw counterparts. From a nutritional standpoint, it is well known that GF foodstuffs are poorer in minerals and protein content, while their saturated fat amount result to be higher compared to their gluten-containing counterparts [41]. TN included in GF free snacks production may represent a valuable alternative when it comes to cover these nutritional deficiencies. Moreover, the lipidic profile mostly characterized by polyunsaturated fatty acids [42], may help to reduce the quantity of the saturated ones. Although, its high fat content imposes a correct evaluation when deciding to employ TN in GF food product development.

### 3.3. Total Soluble Phenolics and Total Antioxidant Capacity of Extruded Snacks

The total soluble phenolic content and the antioxidant capacity were evaluated to assess the nutritional improvement provided by TN (Table 2). The control sample showed low amount of total soluble phenolics, but it had antioxidant activity, which might be related to the reaction of some peptides or amino acids with the DPPH. There was a significant increase in total soluble phenolic and antioxidant capacity when rice flour was replaced with TN in extruded samples. The TSP in the extrudates varied between 10 to 720 µg GAE/mg DW. Adebowale et al. [43] using a cassava-based formulation containing increasing TN, reported a slightly higher TSP (370 to 890 µg GAE/mg DW values), which might be due to the presence of different spices (onion, ginger, chili pepper) in the formulations. TAC results (Table 2) reflected similar trend as that of TSP. The amount of TAC significantly (*p* < 0.01) increased with addition of TN in the extruded products. Those increases may be related with the presence, in the tiger nut cell wall, of some antioxidant monomeric phenols, such as p-hydroxybenzoic acid, vanillic acid, p-hydroxybenzaldehyde, vanillin, p-trans-coumaric acid, trans-ferulic acid, p-cis-coumaric acid, cis-ferulic acid that may contribute to improve the antioxidant capacity [42]. Another possible explanation may come from the presence of tocopherols in TN that have been described as the most important natural group of antioxidants found in vegetable oils [44].

## 4. Conclusions

This study presents the potential use of TN to produce novel gluten-free extruded snacks, providing an attractive alternative to consumers with celiac disease. Pasting profile analysis showed that TN inclusion, into the rice-based formulated flours, increased the onset temperature and delayed the peak viscosity while in the extruded flours. Progressive addition of TN in the formulations promoted a reduction in diameter, expansion ratio, true density, and total pore volume in the extrudates, while their bulk densities increased. Furthermore, TN incorporation was responsible for an increase in ash, protein, and total phenol content, which is an added value to the developed snack. This study demonstrated that extrudates with 10% TN in the formulation showed the best overall texture profile. A future study is proposed using specialty starches to further promote expansion of the TN-based snacks.

## Figures and Tables

**Table 1:** Proximate composition and apparent viscosity of raw blends containing increasing concentrations of tiger nut flour (R1: 10%; R2: 30%; R3: 50%; R4: 70%).

Parameters	Raw Blends				
	Control R	R1	R2	R3	R4
**Proximate Composition**					
Moisture (g/100 g)	11.76 ± 0.12 ^e^	11.52 ± 0.06 ^d^	10.66 ± 0.09 ^c^	9.58 ± 0.08 ^b^	8.72 ± 0.04 ^a^
Protein (g/100 g)	5.02 ± 0.07 ^a^	5.34 ± 0.08 ^b^	5.71 ± 0.12 ^c^	6.08 ± 0.10 ^d^	6.30 ± 0.03 ^e^
Fat (g/100 g)	0.28 ± 0.01 ^a^	3.46 ± 0.08 ^b^	9.06 ± 0.00 ^c^	15.08 ± 0.06 ^d^	20.45 ± 0.19 ^e^
Ash (g/100 g)	0.36 ± 0.01 ^a^	0.51 ± 0.00 ^b^	0.80 ± 0.00 ^c^	1.15 ± 0.03 ^d^	1.46 ± 0.02 ^e^
Carbohydrates (g/100 g)	82.58	79.17	73.77	68.11	63.07
**Apparent Viscosity**					
Onset temperature (°C)	70.3 ± 0.9 ^a^	70.9 ± 0.8 ^a^	71.9 ± 0.4 ^a^	75.1 ± 1.3 ^b^	78.2 ± 1.1 ^b^
Peak Time (min)	5.7 ± 0.0 ^a^	5.8 ± 0.1 ^a^	5.8 ± 0.0 ^a^	5.8 ± 0.1 ^a^	7.1 ± 0.0 ^b^
Peak viscosity (mPa·s)	3980 ± 70 ^e^	3040 ± 60 ^d^	1630 ± 20 ^c^	710 ± 10 ^b^	290 ± 0 ^a^
Trough viscosity (mPa·s)	2130 ± 30 ^e^	1750 ± 100 ^d^	1080 ± 10 ^c^	630 ± 0 ^b^	280 ± 0 ^a^
Breakdown (mPa·s)	1850 ± 40 ^e^	1300 ± 40 ^d^	550 ± 10 ^c^	90 ± 10 ^b^	10 ± 0 ^a^
Final viscosity (mPa·s)	3140 ± 190 ^d^	2900 ± 100 ^d^	2100 ± 0 ^c^	1400 ± 20 ^b^	770 ± 10 ^a^
Total Setback (mPa·s)	1010 ± 220 ^bc^	1160 ± 0 ^bc^	1030 ± 10 ^c^	780 ± 10 ^b^	480 ± 10 ^a^

Means with different letters within the same parameter differ significantly (*p* < 0.01).

**Table 2:** Main physical and nutritional characteristics of extruded samples made from formulations with increasing concentrations of tiger nut flour (E1: 10%; E2: 30%; E3: 50%; E4: 70%).

Parameters	Extruded Samples				
	Control E	E1	E2	E3	E4
**Apparent Viscosity**					
Onset temperature (°C)	50.5 ± 0.0 ^a^	50.4 ± 0.1 ^a^	70.4 ± 0.5 ^b^	79.9 ± 2.5 ^c^	87.2 ± 0.6 ^d^
Peak Time (min)	2.2 ± 0.0 ^a^	4.2 ± 0.2 ^b^	6.5 ± 0.2 ^c^	6.2 ± 0.0 ^c^	7.15 ± 0.1 ^d^
Peak viscosity (mPa·s)	100 ± 10 ^a^	150 ± 0 ^b^	300 ± 0 ^d^	510 ± 10 ^e^	210 ± 0 ^c^
Trough viscosity (mPa·s)	50 ± 0 ^a^	100 ± 0 ^b^	290 ± 0 ^d^	480 ± 10 ^e^	210 ± 0 ^c^
Breakdown (mPa·s)	50 ± 0 ^e^	40 ± 0 ^d^	10 ± 0 ^b^	20 ± 0 ^c^	0 ± 0 ^a^
Final viscosity (mPa·s)	80 ± 0 ^a^	170 ± 0 ^b^	680 ± 0 ^d^	1160 ± 10 ^e^	580 ± 0 ^c^
Total Setback (mPa·s)	30 ± 0 ^a^	70 ± 0 ^b^	390 ± 0 ^d^	670 ± 0 ^e^	370 ± 0 ^c^
**Quality Parameters**					
Diameter (mm)	10.19 ± 0.28 ^d^	9.27 ± 0.27 ^c^	3.46 ± 0.12 ^b^	2.53 ± 0.03 ^a^	2.51 ± 0.03 ^a^
Expansion ratio	16.63 ± 0.92 ^d^	13.78 ± 0.81 ^c^	1.92 ± 0.14 ^b^	1.02 ± 0.02 ^a^	1.01 ± 0.02 ^a^
Bulk density (g/cm³)	0.19 ± 0.02 ^a^	0.21 ± 0.01 ^a^	0.62 ± 0.01 ^b^	0.69 ± 0.02 ^c^	0.65 ± 0.02 ^b^
True density (g/cm³)	1.52 ± 0.01 ^d^	1.54 ± 0.01 ^e^	1.50 ± 0.01 ^c^	1.44 ± 0.01 ^b^	1.35 ± 0.00 ^a^
Total Pore volume (cm³/g)	0.34 ± 0.01 ^d^	0.35 ± 0.00 ^e^	0.33 ± 0.01 ^c^	0.30 ± 0.01 ^b^	0.26 ± 0.00 ^a^
Firmness (g)	712 ± 146 ^c^	922 ± 261 ^d^	242 ± 52 ^a^	367 ± 39 ^b^	313 ± 73 ^ab^
Work of Shear (g·s)	11 ± 3 ^b^	19 ± 6 ^c^	2 ± 1 ^a^	4 ± 1 ^a^	4 ± 1 ^a^
**Proximate Composition**					
Moisture (g/100 g)	6.67 ± 0.07 ^b^	6.12 ± 0.04 ^a^	6.14 ± 0.06 ^a^	6.20 ± 0.05 ^a^	6.24 ± 0.02 ^a^
Protein (g/100 g)	4.65 ± 0.13 ^a^	4.87 ± 0.07 ^b^	5.25 ± 0.09 ^c^	5.57 ± 0.06 ^d^	5.83 ± 0.04 ^e^
Fat (g/100 g)	0.05 ± 0.01 ^a^	1.47 ± 0.01 ^b^	6.62 ± 0.02 ^c^	14.11 ± 0.04 ^d^	19.32 ± 0.14 ^e^
Ash (g/100 g)	0.36 ± 0.00 ^a^	0.67 ± 0.02 ^b^	0.93 ± 0.01 ^c^	1.22 ± 0.02 ^d^	1.52 ± 0.01 ^e^
Carbohydrates (g/100 g)	88.26	86.87	81.07	72.90	67.09
**Total Soluble Phenolics Gallic acid Equivalent (µg/mg)**	10 ± 0.00 ^a^	110 ± 0.00 ^b^	240 ± 0.01 ^c^	550 ± 0.02 ^d^	720 ± 0.03 ^e^
**Antioxidant Capacity Trolox Equivalent (µg/g)**	533 ± 17 ^a^	596 ± 15 ^b^	730 ± 10 ^c^	852 ± 10 ^d^	937 ± 9 ^e^

Means with different letters within the same parameter differ significantly (*p* < 0.01).
